# Reciprocal connectivity between mitral cells and external plexiform layer interneurons in the mouse olfactory bulb

**DOI:** 10.3389/fncir.2013.00032

**Published:** 2013-03-01

**Authors:** Longwen Huang, Isabella Garcia, Hsin-I Jen, Benjamin R. Arenkiel

**Affiliations:** ^1^Department of Neuroscience, Baylor College of Medicine, Texas Children's HospitalHouston, TX, USA; ^2^Program in Developmental Biology, Baylor College of Medicine, Texas Children's HospitalHouston, TX, USA; ^3^Medical Scientist Training Program, Baylor College of Medicine, Texas Children's HospitalHouston, TX, USA; ^4^Department of Molecular and Human Genetics, Baylor College of Medicine, Texas Children's HospitalHouston, TX, USA; ^5^Jan and Dan Duncan Neurological Research Institute, Baylor College of Medicine, Texas Children's HospitalHouston, TX, USA

**Keywords:** olfactory, mitral, interneuron, CRH, circuit, optogenetics, plexiform, synapse

## Abstract

Proper brain function relies on exquisite balance between excitation and inhibition, where inhibitory circuits play fundamental roles toward sculpting principle neuron output and information processing. In prominent models of olfactory bulb circuitry, inhibition of mitral cells by local interneurons sharpens odor tuning and provides contrast enhancement. Mitral cell inhibition occurs at both mitral cell apical dendrites and deep-layer dendrodendritic synapses between granule cells, the most abundant population of inhibitory interneurons in the olfactory bulb. However, it remains unclear whether other local interneurons make inhibitory connections onto mitral cells. Here, we report a novel circuitry with strong and reciprocal connectivity between a subpopulation of previously uncharacterized Corticotropin-Releasing Hormone (CRH)-expressing interneurons located in the external plexiform layer (EPL), and mitral cells. Using cell type-specific genetic manipulations, imaging, optogenetic stimulation, and electrophysiological recordings, we reveal that CRH-expressing EPL interneurons strongly inhibit mitral cell firing, and that they are reciprocally excited by fast glutamatergic mitral cell input. These findings functionally identify a novel subpopulation of olfactory bulb interneurons that show reciprocal connectivity with mitral cells, uncovering a previously unknown, and potentially critical player in olfactory bulb circuitry that may influence lateral interactions and/or facilitate odor processing.

## Introduction

Olfaction plays an essential role in a variety of evolutionarily conserved processes, including mate attraction, aggression, parental behaviors, food recognition, and fear response (Ache and Young, 2005; Dulac and Wagner, 2006). In the mammalian olfactory system, the main olfactory bulb (MOB) receives input from olfactory sensory neurons in the nasal epithelium (Ressler et al., 1994), and sends its output to target cells that reside in higher brain areas (Haberly, 2001; Davison and Ehlers, 2011; Ghosh et al., 2011; Miyamichi et al., 2011; Sosulski et al., 2011). Mitral cells, the excitatory principal neurons in the MOB, are the primary cell types that pass volatile olfactory information from the nasal epithelium to higher cortical regions for processing. Prior to cortical relay, mitral cell output is first sculpted by local interneurons that reside within the olfactory bulb. Initially, periglomerular cells and short-axon cells mediate intraglomerular and interglomerular inhibition at mitral cell apical dendrites (Aungst et al., 2003; McGann et al., 2005; Shao et al., 2012). Granule cells (GCs), the most abundant population of GABAergic interneurons within the MOB, provide a source of deep-layer mitral cell inhibition (Isaacson and Strowbridge, 1998; Schoppa et al., 1998; Chen et al., 2000; Shepherd et al., 2004; Arevian et al., 2008). Residing in the deepest layer of the olfactory bulb, GCs project their dendrites to the external plexiform layer (EPL) and form dendrodendritic synapses onto the lateral dendrites of mitral cells (Price and Powell, 1970a,b). Through dendrodendritic signaling, glutamate release from mitral cells excites GCs, which in turn triggers release of GABA back onto mitral cells. This reciprocal circuitry has inspired models of lateral inhibition, and has been proposed to narrow receptive fields, synchronize the timing of mitral cell firing, and provide olfactory perceptual contrast enhancement (Yokoi et al., 1995; Laurent, 1999; Luo and Katz, 2001; Schoppa and Urban, 2003; Wilson and Mainen, 2006; Fantana et al., 2008; Tan et al., 2010).

Although the general MOB circuitry has been extensively studied (Shepherd et al., 2004), the cell type diversity in the MOB suggests that other key players may also contribute to the currently established model of olfactory bulb circuitry (Pressler and Strowbridge, 2006; Kosaka and Kosaka, 2007; Parish-Aungst et al., 2007; Batista-Brito et al., 2008; Eyre et al., 2008). Notably, in addition to GCs, other populations of local GABAergic interneurons have been described that reside in the EPL and form synapses with the MOB circuitry (Toida et al., 1994; Hamilton et al., 2005; Batista-Brito et al., 2008; Kosaka and Kosaka, 2008, 2011; Lepousez et al., 2010b; Arenkiel et al., 2011). Ultrastructural analyses have shown that subpopulations of parvalbumin- and somatostatin-expressing EPL interneurons form dendrodendritic synapses with mitral/tufted cells (Toida et al., 1994; Lepousez et al., 2010b). However, the functional connectivity between these different populations of EPL interneurons and mitral cells remains unknown.

In the present study, we describe a previously uncharacterized subpopulation of Corticotropin Releasing Hormone (CRH)-expressing interneurons that reside exclusively in the EPL of the MOB. Using a *CRH-Cre* mouse line, we targeted this subset of EPL interneurons for genetic lineage analysis and conditional Channelrhodopsin-2 (ChR2) expression. Employing transgenic and conditional ChR2 virus expression, we manipulated the activity of mitral cells to determine if these two populations of neurons shared functional connectivity. Through cell type-specific activity manipulations, optogenetic stimulation, and electrophysiological recordings, we show that CRH-expressing EPL interneurons make inhibitory connections onto mitral cells, and that they are excited by fast excitatory input from mitral cells. Together these data reveal a novel form of strong and reciprocal feedback circuitry in the MOB.

## Materials and methods

### Experimental mouse lines

Animals were treated in compliance with the US Department of Health and Human Services and Baylor College of Medicine IUCAC guidelines. *CRH-Cre*^+/−^ mice (*Crh*^*tm1(cre)Zjh*^) (Taniguchi et al., 2011) and floxed-conditional *ROSA26 ChR2* mice (*Gt(ROSA)26Sor*^*tm27.1(CAG−COP4 × H134R/tdTomato)Hze*^) were obtained from Jackson Laboratories. *CRH-Cre*^+/−^; *ROSA-lox-stop-lox-ChR2-YFP* mice were generated by crossing male *CRH-Cre*^+/−^ and female *ROSA-lox-stop-lox-ChR2-YFP* mice. *Thy1-ChR2* and *ROSA-lox-stop-lox-tdTomato* mice were previously described (Arenkiel et al., 2007, 2011; Wang et al., 2007). Transgenic *PCDH21-Cre* mice were a kind gift from Mineto Yokoi. In *PCDH21-Cre* mice, the expression of Cre recombinase is controlled by a ~10-kb fragment immediately upstream of the putative translation initiation site of the mouse *PCDH21* gene, and is selectively expressed in mitral/tufted cells in the MOB (Nagai et al., 2005).

### Virus injections

Adeno-Associated Viruses (AAV) serotype 2/9 encoding flexed ChR2 and flexed tdTomato (*AAV2/9.EF1a.DIO.hChR2(H134R)-EYFP.WPRE.hGH* and *AAV2/9.CAG.FLEX.tdTomato.WPRE.bGH*) plasmid constructs were obtained from the University of Pennsylvania Vector Core and packaged in house. For CRH+ EPL interneuron morphology analysis, 50 nL AAV (2.5 × 10^12^ viral particles/mL) was injected into the MOB (from bregma: *ML*, ± 0.9 mm; AP, 3.82 mm; and 0.1 mm down from the surface of the MOB) of *CRH-Cre* mice using glass injection pipettes and a Nanoject II (Drummund Scientific Company, Broomall, PA) at a rate of 23 nl/s at 20 s intervals. At 10–14 d post-injection, the animals were deeply anesthetized using isoflurane, and perfused intracardially using 4% paraformaldehyde (PFA). Brains were dissected, post-fixed overnight, and the olfactory bulbs were sliced for imaging. For the mitral cell—EPL interneuron connectivity experiments, 500 nL AAV was injected into the core of the MOB (from bregma: ML, ±0.9 mm; AP, 3.82 mm; DL, −2.88 mm) of *PCDH21-Cre* mice. The olfactory bulbs were dissected and sliced for imaging or electrophysiology at 10–14 d post-injection. For mitral cell—CRH+ EPL interneuron connectivity experiments, 500 nL AAV (flexed tdTomato) was injected into the MOB (from bregma: ML, ±0.9 mm; AP, 3.82 mm; and 0.1 mm down from the surface of the MOB) of *Thy1-ChR2; CRH-Cre* mice. Olfactory bulbs were dissected and sliced for electrophysiology at 12–14 d post-injection.

### Immunohistochemistry, histology, and imaging

For immunohistochemistry, animals were deeply anesthetized using isoflurane, followed by intracardial perfusion of PBS and 4% PFA. Brains were dissected and post-fixed in 4% PFA for 1 h at room temperature or overnight at 4°C. Olfactory bulbs were sectioned at 50 μm using a Compresstome (Precisionary Instruments, San Jose, CA) and incubated in blocking solution (10% normal goat serum, 0.3% Triton X-100 in PBS, pH 7.35) at 4°C overnight. Sections were stained using rabbit anti-CRH (kindly provided by Nicholas Justice, Baylor College of Medicine), rabbit anti-Calretinin (1:1000, Millipore AB5054), mouse anti-GFAP (1:1000, NeuroMab, UC Davis), mouse anti-NeuN (1:1000, Millipore MAB377), rabbit anti-Somatostatin (1:250, Immunostar 3C11), guinea pig anti-Parvalbumin (1:200, Synaptic Systems 195004), rabbit anti-Tyrosine Hydroxylase (1:2000, Chemicon Ab152), or rabbit anti-βIV-spectrin (kindly provided by Matthew Rasband, Baylor College of Medicine). Primary antibodies were diluted in blocking solution and applied overnight at 4°C. The next day, olfactory bulb slices were washed 4 × 10 min each in PBS with 0.1% Triton X-100. Secondary Alexa-488 anti-rabbit, mouse, or guinea pig IgG (Invitrogen, Carlsbad, CA) were used at a final dilution of 1:500 and incubated for 1 h at room temperature. Slices were washed 4 × 15 min each and mounted with Vectashield mounting medium containing DAPI (Vector Laboratories, Burlingame, CA). Imaging was performed using a Leica TCS SPE confocal microscope under a 20× objective. Neuronal marker expression was quantified by analyzing 180 × 180 × 10 μm^3^ fields of view and is reported as percentage ± SEM (*n* = 3 animals each, 5 slices per animal, 5 sections per slice). Whole bulb images of *CRH-Cre; lox-stop-lox-tdTomato*, *CRH-Cre; lox-stop-lox-ChR2-YFP*, and *PCDH21-Cre*; AAV-flexed ChR2 mice were taken on a Leica M205FA stereo-dissection microscope.

For cell morphology analysis after biocytin injection, the recorded slices were fixed in 4% PFA at 4°C overnight. The following day, slices were washed 5 min in PBS, incubated in 0.1% H_2_O_2_ for 10 min to quench endogenous peroxidases, washed 2 × 5 min in PBS, and incubated in ABC working solutions (Vectastain ABC Kit, Vector Laboratories, Burlingame, CA) with 0.1% Tween at 4°C overnight. After washing the slices 4 × 5 min in PBS, DAB solution (0.25 mg/mL, with 0.007% H_2_O_2_) was applied for 2 min. Signal development was quenched and the tissue was dehydrated in serially diluted ethanol, and mounted using permount mounting medium. Images were taken using a Zeiss Axioimager Z1 microscope under a 40× objective lens. Morphology analyses were performed using Neurolucida software (MBF Bioscience, Williston, VT).

### Acute brain slice preparation and electrophysiology

Coronal olfactory bulb slices (300 μm) were prepared from either *CRH-Cre; lox-stop-lox-ChR2-YFP* mice (P42-P56), *Thy1-ChR2* mice (P21-P35), *PCDH-Cre*; AAV flexed ChR2 mice (P49–P56, 12 dpost-injection), *CRH-Cre; Thy1-ChR2;* AAV flexed tdTomato mice (P42–P56, 14 dpost-injection), or *CRH-Cre; lox-stop-lox-tdTomato* mice (P21–P35). Animals were deeply anesthetized using isoflurane, and perfused intracardially with ice-cold artificial cerebrospinal fluid (ACSF, in mM: 122 NaCl, 3 KCl, 1.2 NaH_2_PO_4_, 26 NaHCO_3_, 20 glucose, 2 CaCl_2_, 1 MgCl_2_, 305–310 mOsm, pH 7.3). Brains were dissected and rapidly embedded in low melting point agarose and sectioned into ice-cold oxygenated (5% CO_2_, 95% O_2_) dissection buffer (in mM: 87 NaCl, 2.5 KCl, 1.6 NaH_2_PO_4_, 25 NaHCO_3_, 75 sucrose, 10 glucose, 1.3 ascorbic acid, 0.5 CaCl_2_, 7 MgCl_2_), recovered (15 min at 37°C) in oxygenated artificial ACSF, and acclimated at room temperature for 10 min prior to electrophysiological recordings.

Borosilicate glass electrodes (Sutter Instruments, Novato, CA) were used for whole cell patch clamp recordings. Electrodes were pulled with tip resistance between 4 and 7 MΩ, and filled with internal solution (for current clamp: in mM, 120 K-gluconate, 5 KCl, 2 MgCl_2_, 0.05 EGTA, 10 HEPES, 2 Mg-ATP, 0.4 Mg-GTP, 10 creatine phosphate, 290–300 mOsm, pH 7.3; for voltage clamp: in mM, 89 CsMeSO_3_, 46 CsCl, 1 MgCl_2_, 0.16 CaCl_2_, 0.2 EGTA, 15 HEPES, 4 Na-ATP, 0.4 Na-GTP, 15 TEA-Cl, 14 creatine phosphate, 290–300 mOsm, pH 7.3). During recordings, coronal olfactory bulb slices were placed in a RT chamber mounted on an Olympus upright microscope (BX50WI) and perfused with oxygenated ACSF. Cells were visualized under differential interference contrast imaging. Data were obtained via a Multiclamp 700B amplifier, low-pass Bessel filtered at 4 kHz, and digitized on computer disk (Clampex, Axon Instruments). Excitation light was filtered by an EGFP filter, and controlled by a Smart Shutter Controller (Sutter Instruments, Novato, CA), which received digital commands from Clampex, to activate ChR2-expressing neurons in brain slices. The average light intensity was measured to be ~20 mW/mm^2^ using a PM20 optical power meter (Thorlabs, Inc., Newton, NJ), and the illuminating area was ~3 mm^2^. Bicuculline (50 μM), APV (20 μM), CNQX (20 μM), GABAzine (12.5 μM), and TTX (1 μM) were purchased from Sigma-Aldrich, and bath-applied to the recording chamber during electrophysiological recordings. For biocytin injections, 8 mM biocytin was added to the internal solution (current clamp), cells were held at resting potentials for 5 min, and the pipette was carefully retracted. No liquid-junction potential was corrected. The statistical analyses were performed using two-tailed Student's *t*-test for 2-group comparison.

### *In vivo* photostimulation, odor presentation, and electrophysiology

*In vivo* recordings were performed on animals anesthetized with ketamine/dexdormatore (75 mg/kg) via intraperitoneal injection, followed by sustained 0.3% isoflurane with oxygen delivery to the animal. The dorsal surface of the olfactory bulb was carefully exposed as to not damage the pia or underlying brain tissue. Cells were confirmed as mitral cells based on recording depth, coupled baseline firing to respiration, and background activity in the mitral cell layers (Arenkiel et al., 2007). For light stimulation and electrophysiological recordings, fiber optics and 1.0 MΩ extracellular recording electrodes (Microprobe Inc., Gaithersburg, MD) were used. A blue laser source (CrystaLaser, Reno, NV), was controlled by a Master-8 (A.M.P.I., Israel), and guided to the olfactory bulb by focusing light onto fused silica fiber optics. The average light intensity was measured to be ~30 mW/mm^2^. Extracellular recordings were amplified using a Model 1800 AC amplifier (A-M systems, Carlsberg, WA), digitized by CED Power 1401 mk II, and processed using Spike2 acquisition software (Cambridge Electronic Design, Cambridge, England). Statistical analyses were performed using two-tailed paired Student's *t*-test for 2-group comparison.

During *in vivo* recordings, odors were presented using a liquid-dispensing robot (F5200, I & J Fisnar, Fair Lawn, NJ) coupled to a Master-8 (A.M.P.I., Israel) and a picospritzer (Science Products, Germany) for odor presentation. The odorant mixture used was a solution containing 1% each of methyl salicylate, trans-cinnamaldehyde, amyl acetate, pinene, citral, and limonene (each obtained from Sigma) in mineral oil. The odorized air was delivered into the continuous oxygen stream controlled via a Master-8.

## Results

### *CRH-Cre* targets a subset of EPL interneurons in the olfactory bulb

**Figure 1:**
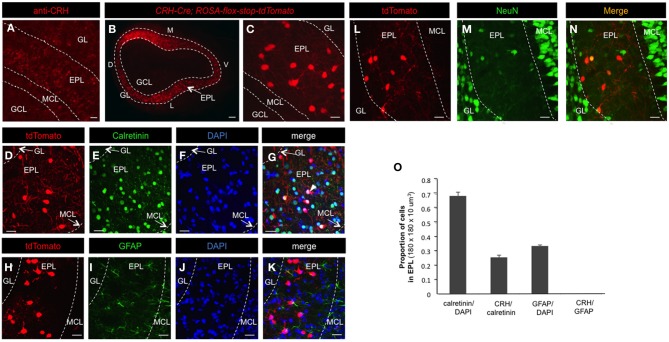
**CRH-Cre targets a subset of EPL interneurons. (A)** Confocal image highlighting CRH protein expression in the MOB. Scale bar, 60 μm. **(B)** Epifluorescent image showing conditional tdTomato expression in the olfactory bulb of *CRH-Cre; ROSA-lox-stop-lox-tdTomato* mice. Scale bar, 300 μm. **(C)** High magnification confocal image showing tdTomato expression in EPL. Scale bar, 15 μm. **(D–G)** Confocal image of calretinin and DAPI staining of cells in *CRH-Cre; ROSA-lox-stop-lox-tdTomato* mice. Scale bar, 20 μm. **(H–N)** Confocal image of GFAP and NeuN staining of cells in *CRH-Cre; Rosa-lox-stop-lox-tdTomato mice*. Scale bar, 20 μm. **(O)** The proportion of calretinin+ cells in all EPL cells, CRH+ neurons in all calretinin+ interneurons, GFAP+ cells in all EPL cells, and CRH+ neurons in all GFAP+ cells. *N* = 3 mice, and 20–30 sections each. Error bars represent SEM. GL, glomerular layer; EPL, external plexiform layer; MCL, mitral cell layer; GCL, granule cell layer; D, dorsal; V, ventral; M, medial; L, lateral. All the images are from coronal sections. In panels (**B,C,D,H**, and **L**), the red fluorescence is directly from tdTomato without antibody enhancement.

Neuronal subtypes show extensive molecular and functional diversity, even within regionally restricted and/or morphologically uniform populations of cells. This genetically programmed cellular diversity underlies both anatomical and functional differences between and amongst neural microcircuits, and thus ultimately influences critical aspects of brain processing. To begin to identify uniquely distributed neuronal subtypes that populate the EPL of the mouse MOB, we performed immunohistochemistry using antibodies against diverse interneuron makers. Although most of these markers, including calretinin, somatostatin, and parvalbumin were broadly localized and did not show regionally restricted laminar expression (data not shown), anti-CRH staining revealed an enriched staining pattern in the EPL (Figure 1A). Because CRH is a small, secreted neuropeptide, immunohistochemical staining patterns of this marker have the potential of labeling both CRH-expressing EPL interneurons, as well as extracellularly secreted CRH, thus complicating the ability to definitively identify CRH positive cells. To circumvent this issue, and to more accurately determine the CRH expression pattern in EPL interneurons, we applied genetic labeling methods. For this, we performed genetic lineage analysis by driving conditional red fluorescent protein expression in *ROSA-lox-stop-lox-tdTomato* mice using a *CRH-Cre* knock-in driver line (Taniguchi et al., 2011). In *CRH-Cre; ROSA-lox–stop-lox-tdTomato* mice, the tdTomato reporter was robustly expressed in a subset of EPL interneurons (Figures 1B,C), while interneurons in the glomerular layer (GL), internal plexiform layer (IPL), or granule cell layer (GCL) were not targeted and thus showed no tdTomato expression. Occasionally, a small proportion (less than 5%) of mitral cells showed low tdTomato expression in some *CRH-Cre; ROSA-lox–stop-lox-tdTomato* individuals. However, the fluorescent reporter was consistently and strongly expressed in subsets of EPL interneurons (Figures 1B,C).

**Figure 2:**
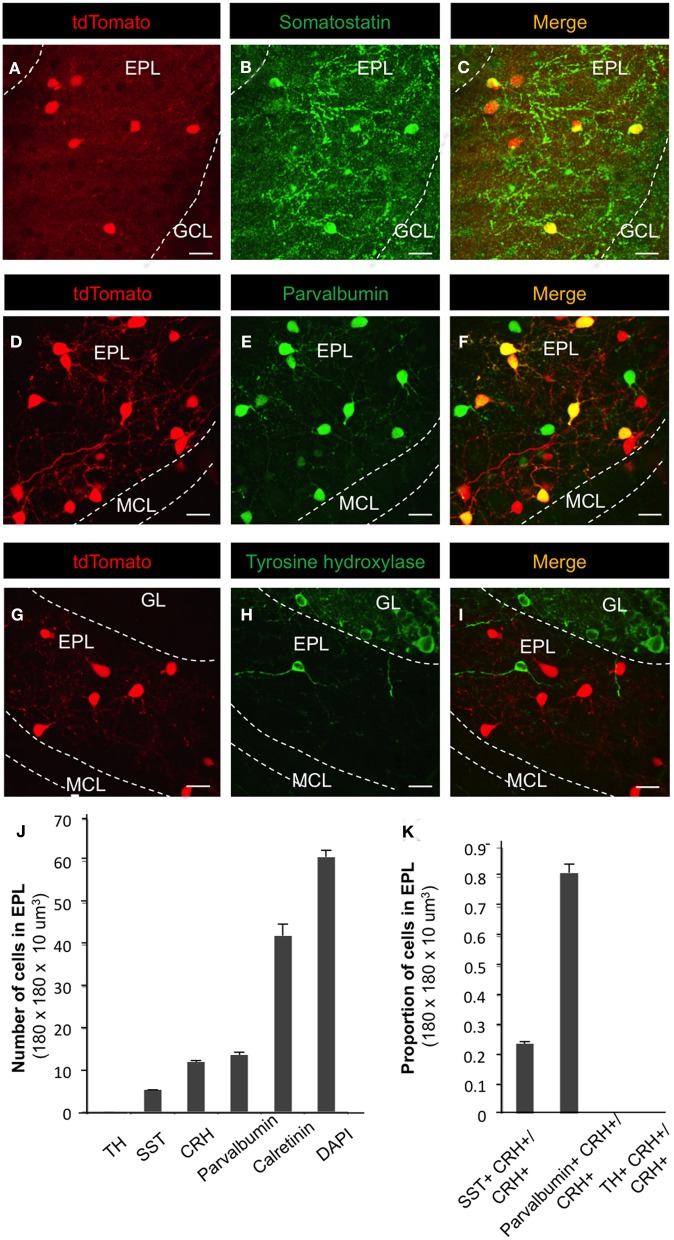
**Molecular marker expression in CRH+ EPL interneurons. (A–I)** Expression patterns of somatostatin, parvalbumin, and tyrosine hydroxylase in *CRH-Cre; Rosa-lox-stop-lox-tdTomato* mice. Scale bar, 20 μm. **(J)** The number of cells that are labeled by tyrosine hydroxylase (TH), somatostatin (SST), CRH, parvalbumin, calretinin, and DAPI in EPL. *N* = 3 mice, and 20–30 sections each. **(K)** The fraction of CRH+ neurons that are doubly positive for somatostatin, parvalbumin, or tyrosine hydroxylase. GL, glomerular layer; EPL, external plexiform layer; MCL, mitral cell layer; GCL, granule cell layer. Marker analysis from *n* = 3 mice, 25 sections each. All the images are from coronal sections. Error bars represent SEM. In panels (**A**,**D**, and **G**), the red fluorescence is directly from tdTomato without antibody enhancement.

Next, we sought to determine the fraction of EPL interneurons that expressed CRH. Toward this, we performed immunohistochemistry and confocal microscopy on MOB tissue from *CRH−Cre; ROSA–flox–stop–flox–tdTomato* mice using molecular markers for various interneuron and glial cell types (Figures 1 and 2). To quantify the relative fraction of CRH-expressing cells from the total number of interneurons that reside in the EPL, we first stained for calretinin, a broadly expressed interneuron marker to estimate the total number of EPL interneurons. We found that all CRH+ neurons are calretinin positive (calretinin+), on average the CRH+ EPL interneurons (doubly labeled tdTomato/calretinin neurons) constituted 25.69 ± 1.31% (*mean* ± SEM, *n* = 3 mice, 25 sections each) of all calretinin+ interneurons, and calretinin+ interneurons constituted 68.75 ± 2.70% of all the EPL cells (as shown by DAPI) (Figures 1D–G,O). To confirm that the CRH+ cells were not glia, we stained for both GFAP and NeuN. We found that there was no overlap between CRH+ cells and GFAP+ cells (Figures 1H–K,O). Interestingly, the expression level of NeuN in CRH+ EPL interneurons was low and sparse (44.2 ± 1.7%) (Figures 1L–N). Together, these data suggest that CRH+ cells are not glial cells, and they are among the few neuron types that lack strong NeuN expression (Mullen et al., 1992). To further characterize the molecular profiles of CRH+ EPL interneurons, we next stained for somatostatin, parvalbumin, and tyrosine hydroxylase, and characterized the overlapping expression in CRH+ interneurons (Figure 2). While tyrosine hydroxylase+ interneurons are completely non-overlapping with CRH+ EPL interneurons, we observed variable levels of somatostatin (24.8 ± 0.8%) and parvalbumin (81.5 ± 3%) coexpression (Figures 2A–K), suggesting a significant level of biochemical heterogeneity amongst EPL interneurons.

**Figure 3:**
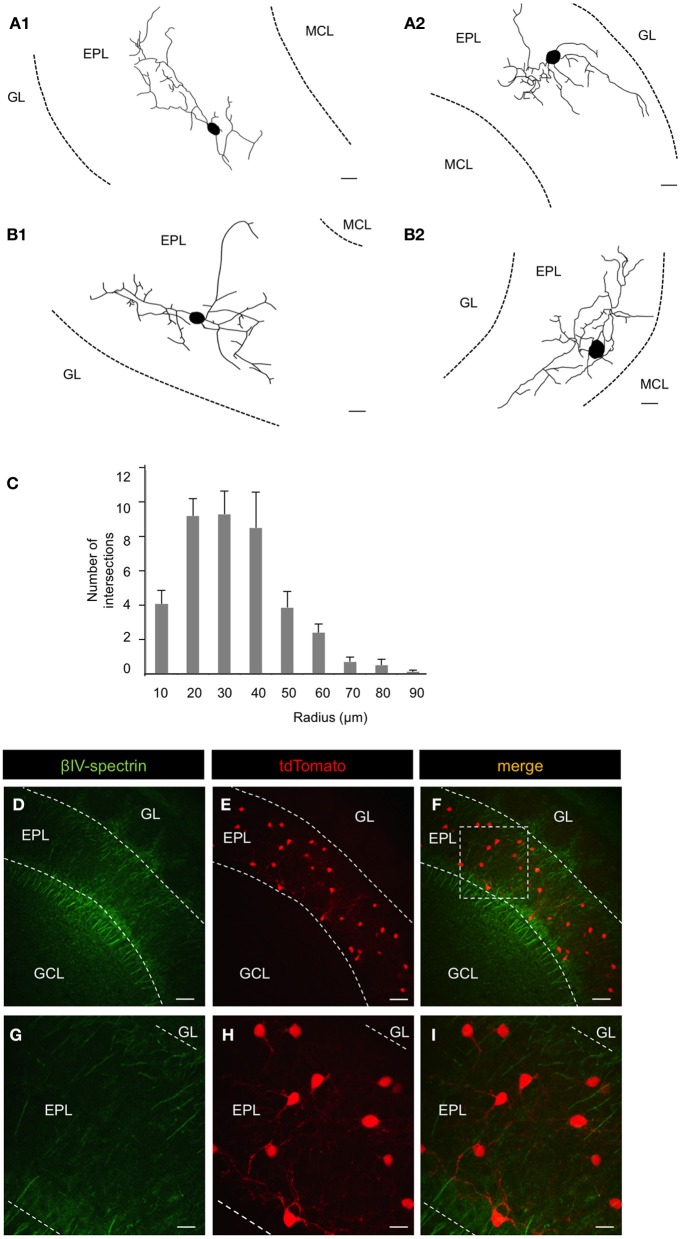
**Morphological and electrical properties of EPL interneurons in the MOB. (A1)** and **(A2)** Reconstruction of CRH+ EPL interneurons labeled via biocytin fill. Scale bar, 10 μm. **(B1)** and **(B2)** Reconstruction of CRH+ EPL interneurons labeled via AAV (flexed ChR2) injection into a *CRH-Cre* mouse. Scale bar, 10 μm. **(C)** Sholl analysis of CRH+ EPL interneurons, *n* = 10. Error bars represent SEM. **(D–I)** β-spectrin staining in *CRH-Cre; ROSA-lox-stop-lox-tdTomato* mice. **(G–I)** are high magnification images from the inset shown in **(F)**. Scale bar **(D–F)**, 50 μm; **(G–I)**, 15 μm. All the images are from coronal sections. Red fluorescence is directly from tdTomato without antibody enhancement.

To determine the morphological properties of individual CRH+ EPL interneurons, we injected biocytin into single CRH+ EPL interneurons in brain slices from *CRH-Cre; ROSA-lox-stop-lox-tdTomato* mice and performed histological staining (Figure 3A). We also injected low-titer AAV encoding flexed ChR2 (Atasoy et al., 2008) into the olfactory bulbs of CRH-Cre mice to achieve sparse membrane labeling of CRH+ interneurons in the MOB (Figure 3B). Both methods revealed similar CRH+ EPL interneuron morphologies, so we pooled morphological data for quantitative analysis. We found that CRH+ EPL interneurons are multipolar cells with 3.5 ± 0.4 primary processes, and an average soma diameter of 9.6 ± 0.7 μm. The neurites spanned up to 71 ± 4.5 μm from the cell body, and sholl analysis showed that the highest amount of dendritic branching occurred within 30 μm from the cell body (*n* = 10, *mean* ± SEM) (Figure 3C). In order to determine the nature of these neuronal processes, we stained the MOB tissues from *CRH−Cre; ROSA–flox–stop–flox–tdTomato* mice with antibodies that recognized the axonal marker βIV-spectrin. By immunohistochemistry and confocal imaging, we found that there was no expression of βIV-spectrin in the CRH+ EPL interneuron neurites (Figures 3D–I), suggesting that CRH+ EPL interneurons are axonless.

**Figure 4:**
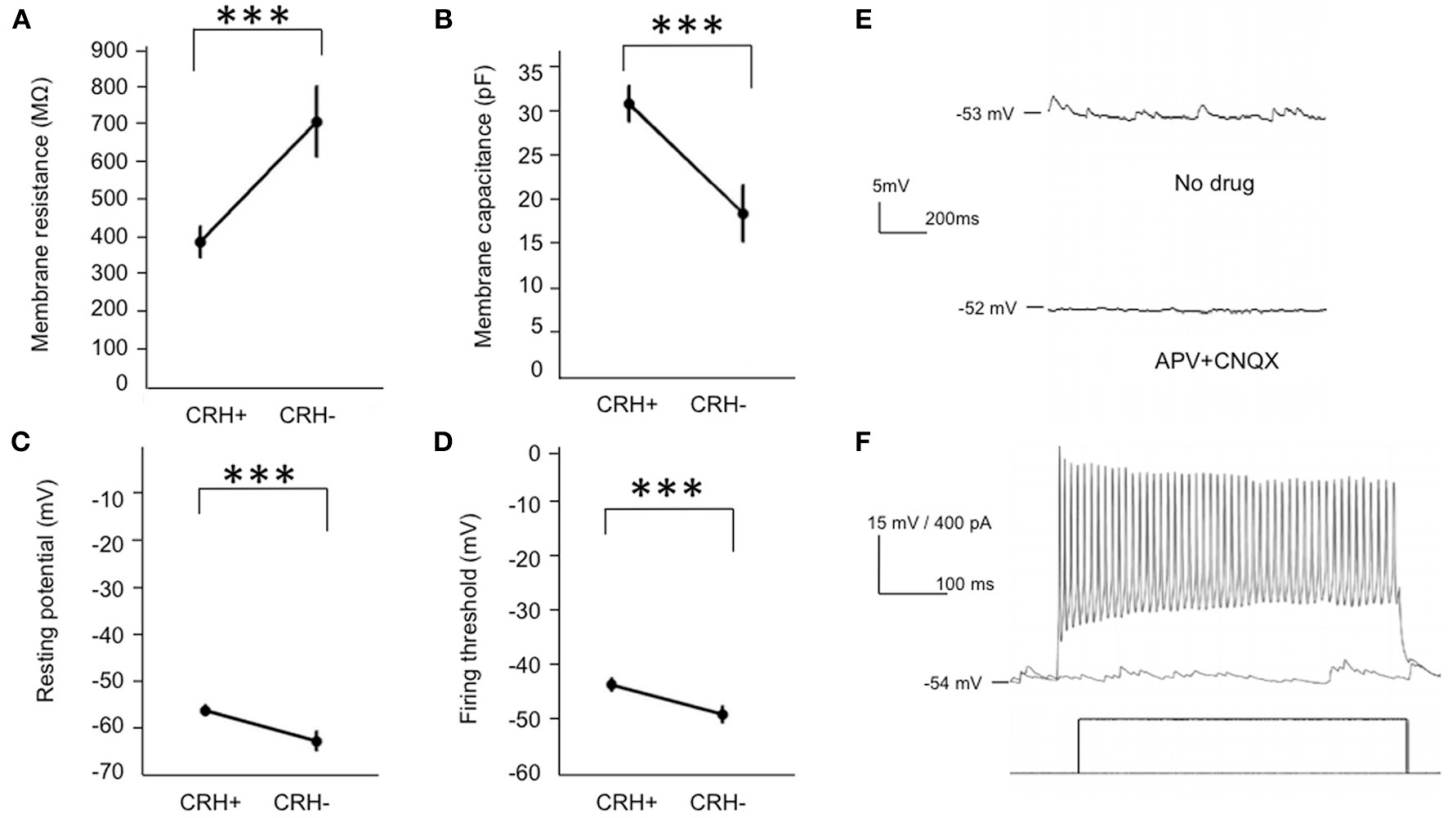
**Electrical properties of EPL interneurons in the MOB. (A–D)** Membrane resistance, membrane capacitance, resting membrane potential, and action potential threshold of CRH+ (*n* = 25) and CRH− EPL (*n* = 10) interneurons. Student's *t*-test, ^***^p < 0.01. Error bars represent SEM. **(E)** Upper, a typical trace showing spontaneous postsynaptic currents in a CRH+ EPL interneuron. Lower, the spontaneous postsynaptic potentials is largely blocked by APV + CNQX. **(F)** A representative trace showing a CRH+ EPL interneuron firing in response to current injection.

To reveal the cell type-specific electrical properties of these EPL interneurons, we next performed whole cell patch clamp recordings in acute brain slices from *CRH-Cre; ROSA–flox–stop–flox–tdTomato* mice, and recorded from both CRH+ EPL interneurons and CRH−negative (CRH−) EPL interneurons. We found that compared to CRH− EPL interneurons, CRH+ EPL interneurons show significantly lower resistance (Figure 4A) and higher capacitance (Figure 4B). Moreover, CRH+ interneurons also showed more depolarized resting membrane potentials (Figure 4C), but higher threshold for action potentials (Figure 4D). CRH+ cells had baseline firing rates of 0.20 ± 0.09 Hz (*n* = 14), and they showed highly active subthreshold activity (EPSP, frequency 11.45 ± 3.62 Hz, amplitude 1.64 ± 0.10 mV, *n* = 14, mean ± SEM), which was almost completely blocked by APV (20 μM) and CNQX (20 μM) (Figure 4E). These cells also exhibited high firing rates in response to current injection, with maximum firing rates of up to 77.20 ± 6.14 Hz (*n* = 10) (Figure 4F). Taken together, these data suggest that CRH-expressing EPL interneurons comprise a population of medium-sized, and fast-spiking interneurons in the olfactory bulb, receive high frequency excitatory input from other neurons, and based on morphological criteria may be capable of mediating interactions between spatially distributed mitral cells.

### Conditional expression of ChR2 allows for cell type-specific manipulation of CRH+ interneuron activity

**Figure 5:**
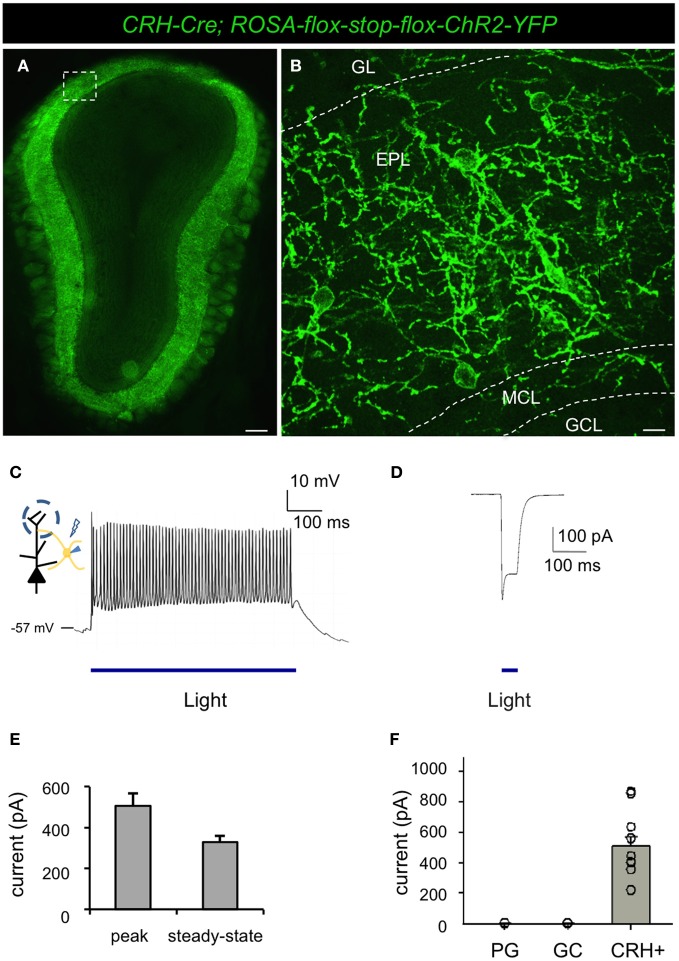
**Conditional expression of ChR2 under the control of CRH-Cre allows optogenetic manipulation of a subset of EPL interneurons. (A)** Epifluorescence microscope image showing conditional ChR2 expression in a subset of EPL interneurons in *CRH-Cre; ROSA-lox-stop-lox-ChR2* mice. **(B)** Confocal image showing a higher magnification view of inset shown in **(A)** highlighting ChR2 localization to the membrane and dendritic processes of EPL interneurons. GL, glomerular layer; EPL, external plexiform layer; MCL, mitral cell layer; GCL, granule cell layer. Scale bars: left, 300 μm; right, 15 μm. **(C)** Whole cell recording showing optogenetic induction of action potentials in EPL interneurons-expressing ChR2. **(D)** Light-evoked current response in ChR2-expressing EPL interneurons. **(E)** The peak and steady-state amplitude of light-evoked currents in EPL interneurons. *n* = 11. Error bars represent SEM. **(F)** Light-evoked currents in ChR2-expressing EPL interneurons in the presence of synaptic blockers. EPL IN, EPL interneuron, *n* = 11; GC, granule cells, *n* = 9; PG, periglomerular cells, *n* = 8. Error bars represent SEM. Images in (**A** and **B**) are from coronal section.

To determine the functional connectivity between neuronal subsets, it is useful to selectively manipulate the activity of targeted neurons. For this, optogenetic approaches afford exquisite control over genetically defined neuronal subtypes (Boyden et al., 2005). To begin to elucidate the functional connectivity of CRH+ EPL interneurons, we crossed male *CRH-Cre* mice to female *ROSA-lox-stop-lox-ChR2-YFP* mice (Madisen et al., 2012) to allow selective and restricted photo control of ChR2 in CRH+ EPL interneurons (Figures 5A,B). Similar to what we observed in the conditional tdTomato background, reporter activation using the CRH-Cre driver resulted in high levels of ChR2 expression in interneuron subsets throughout the EPL (Figures 5A,B). Unlike the red cell fill that was observed following conditional floxed tdTomato expression, ChR2-YFP localized to neuronal membranes, highlighting the extensive dendritic arborizations of EPL interneurons within the bulb (Figure 5B). In order to determine the light responsiveness of ChR2-expressing neurons, we performed whole cell patch clamp recordings from CRH+ EPL interneurons in acute olfactory bulb slices that were subjected to photostimulation. We found that long-pass filtered blue light from a xenon light source efficiently and robustly evoked action potentials in CRH+ EPL interneurons (Figure 5C), with a maximum firing rate of 77.93 ± 10.70 Hz (*n* = 11, mean ± SEM), which is close to the maximum firing rate we observed by current injection (see above). Brief periods of shutter-controlled photostimulation generated precisely timed photocurrents with average amplitudes of 506.1 ± 61.8 pA (peak), and 330.4 ± 30.7 pA (steady-state) (Figures 5D,E). Time latency from light initiation to the peak of the first action potential was 7.76 ± 0.47 ms (*n* = 11). To validate the cellular specificity of the CRH-Cre line, we also made recordings under blue light from other local interneurons in the olfactory bulb, including granule cells and periglomerular cells. In the presence of synaptic blockers (CNQX 20 μM, APV 20 μM, and Bicuculline 50 μM), we found that only CRH+ EPL interneurons showed direct light-evoked currents (Figure 5F), which is consistent with imaging data showing that *CRH-Cre* only targets a subset of EPL interneurons (Figures 1B,C).

### EPL interneurons make inhibitory connections onto mitral cells

**Figure 6:**
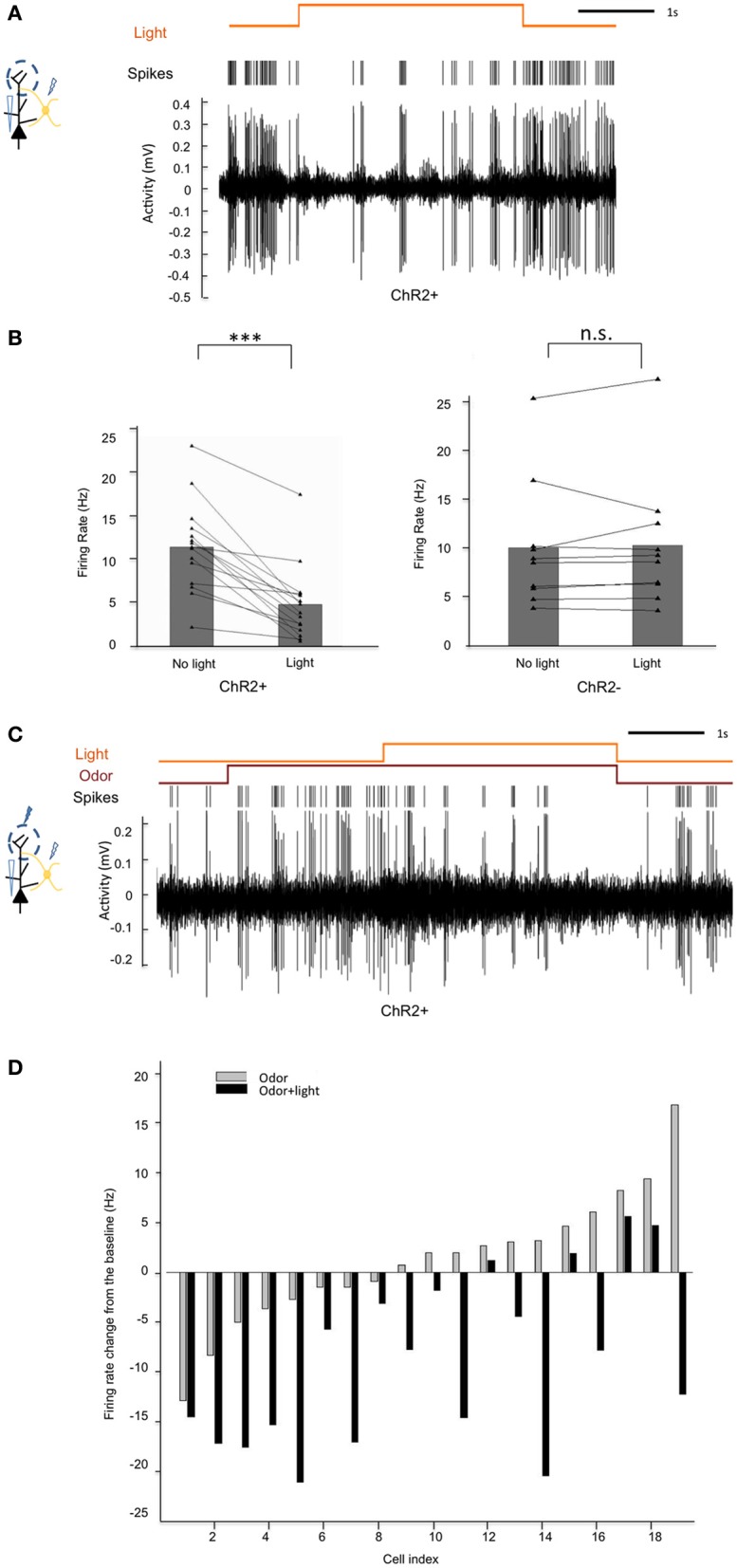
**Activation of CRH+ EPL interneurons inhibits spontaneous and odor-evoked mitral cell firing *in vivo*. (A)** A representative trace showing the inhibition of spontaneous mitral cell firing by light activation of CRH+ EPL interneurons during extracellular single unit recording in a *CRH-Cre; Rosa-lox-stop-lox-ChR2* mouse. **(B)** Statistical analysis showing rate changes of spontaneous mitral cell firing in response to blue light in ChR2+ (left) and ChR− (right) mice. In ChR2+ mice, spontaneous mitral cell firing was significantly decreased when CRH+ EPL interneurons are photo-activated, *N* = 20. Student's test, ^***^p < 0.01. However, in ChR− mice, light stimulation had no significant effect, *N* = 10. Student's *t*-test, n.s., *p* > 0.05. **(C)** A representative trace showing mitral cell excitation by an odorant mixture, and subsequent inhibition of odor-evoked firing following CRH+ EPL photostimulation. **(D)** Group analysis showing how 19 different mitral cells responded to an odorant mixture, and how the responses were affected by light activation of CRH+ EPL interneurons. The bars show the changes of mitral cell firing from baseline (no odor, no light).

Having shown the ability to specifically manipulate the activity of CRH+ EPL interneurons, we next sought to identify the nature of synaptic connectivity of these neurons within the local MOB circuitry. To determine the influence of EPL interneuron activity on mitral cell firing, we first performed *in vivo* extracellular recordings from mitral cells while optically manipulating CRH+ EPL interneuron activity in anesthetized *CRH-Cre; ROSA-lox-stop-lox-ChR2-YFP* mice, both in the absence and presence of odor stimuli. In the spontaneous firing state, we observed significant inhibition of mitral cell firing in response to blue light in the experimental mice, but no significant change of mitral cell firing in C57Bl/6 wild type mice, suggesting that light-activated GABAergic EPL interneurons inhibited mitral cells (Figures 6A,B). We then presented an odorant mixture, and paired odor presentation with light stimulation in *CRH-Cre; ROSA-lox-stop-lox ChR2-YFP* mice to determine if CRH+ EPL interneuron activity influenced mitral cell responses to odorant stimuli (Figure 6C). Interestingly, we found that even though mitral cells responded differently (excitation vs. inhibition) to the odorant mixture, activation of CRH+ EPL interneurons consistently showed inhibitory effects on mitral cell responses (Figure 6D). These data demonstrate that EPL interneurons inhibit both spontaneous and odor-evoked activity in mitral cells, suggesting their inhibitory connectivity onto mitral cells.

**Figure 7:**
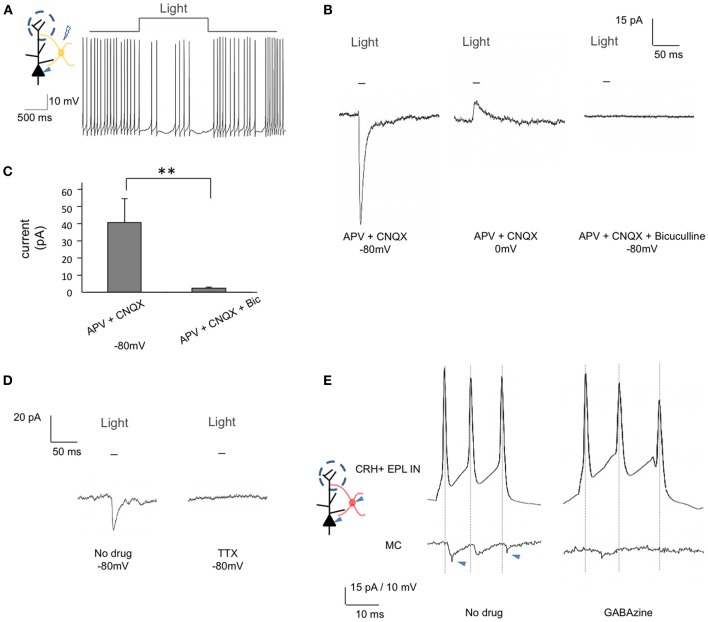
**EPL interneurons make inhibitory connections onto mitral cells. (A)** and **(B)** Whole cell recordings from mitral cells in brain slices from *CRH-Cre; ROSA-lox-stop-lox-ChR2* mice. **(A)** Inhibition of mitral cell firing in response to EPL interneuron photostimulation. **(B)** Recorded GABAergic postsynaptic currents in mitral cells following EPL interneuron photostimulation. The current was inward when the cell was held at −80 mV (left) but changed its direction when the cell was held at 0 mV (middle). Resulting photocurrents are refractory to glutamate receptor blockers, but are absent in the presence of Bicuculline (right). Data shown is the average of 10 repeated traces. **(C)** Average amplitudes of light-evoked postsynaptic currents in mitral cells. Bic, Bicuculline. APV + CNQX, *n* = 10; APV + CNQX + Bicuculline, *n* = 7. Student's *t*-test, ^**^p < 0.025. Error bars represent SEM. **(D)** The recorded postsynaptic current was blocked by TTX. The shown data is the average of 10 repeated traces. **(E)** Unitary connectivity from a CRH+ EPL interneuron to a mitral cell. A short pulse of current was injected into the CRH+ EPL interneuron to evoke action potentials, and postsynaptic current was observed in the mitral cells (left). The postsynaptic currents were fully blocked by GABAzine (right). Jitters indicated by arrowheads are from external noises during the recording.

To further confirm the *in vivo* electrophysiological observations, we next performed whole cell recordings from mitral cells in acute brain slices from *CRH-Cre; ROSA-lox-stop-lox-ChR2-YFP* mice. While recording action potentials from mitral cells, we observed strong and robust inhibitory effects following optogenetic activation of EPL interneurons (Figure 7A). To determine the nature of EPL interneuron—mitral cell connectivity, we next held mitral cells at different potentials and measured light-evoked postsynaptic currents. Mitral cells showed strong postsynaptic currents in response to blue light (41.42 ± 14.29 pA, −80 mV) (*n* = 10, mean ± SEM) (Figures 7B,C), with time latencies from light initiation to the onset of postsynaptic currents of 7.83 ± 0.29 ms (*n* = 10), suggesting that mitral cells receive fast inhibitory input from CRH-expressing EPL interneurons (note that the time latency from light initiation to the peak of CRH+ EPL interneuron action potential was 7.76 ± 0.47 ms, see above). At a holding potential of 0 mV, we observed light-evoked postsynaptic currents in the opposite direction, revealing a GABAergic photo-evoked current (Figure 7B). To further confirm the GABAergic input, we employed pharmacological methods, and found that the postsynaptic currents were fully blocked by 50 μM Bicuculline (2.21 ± 0.74 pA, *n* = 7) (Figures 7B,C). Moreover, bath application of 1 μM TTX also fully blocked light-evoked postsynaptic current in mitral cells, suggesting that postsynaptic currents were action potential dependent (Figure 7D). Together, data from both *in vivo* and *in vitro* recordings show that CRH+ EPL interneurons make strong and robust inhibitory connections onto mitral cells.

To measure the unitary connectivity from CRH+ EPL interneurons to mitral cells, we next performed paired whole cell recordings between genetically labeled CRH+ EPL interneurons and mitral cells. Using *CRH-Cre; lox-stop-lox-tdTomato* mice, we recorded pairs of mitral cells and red EPL interneurons within distances of 80 μm from each other. Of the 17 pairs attempted, 3 pairs showed unitary connectivity (Figure 7E), which was blocked by 12.5 μM GABAzine. The average amplitude of the postsynaptic current was 6.83 ± 1.31 pA, and the time latency from the peak of CRH+ EPL interneuron action potential to the onset of mitral cell postsynaptic current was 1.07 ± 0.26 ms (*n* = 3, mean ± SEM), strongly suggesting monosynaptic connectivity between CRH+ EPL interneurons and mitral cells.

### Mitral cells make excitatory connections onto CRH+ EPL interneurons

**Figure 8:**
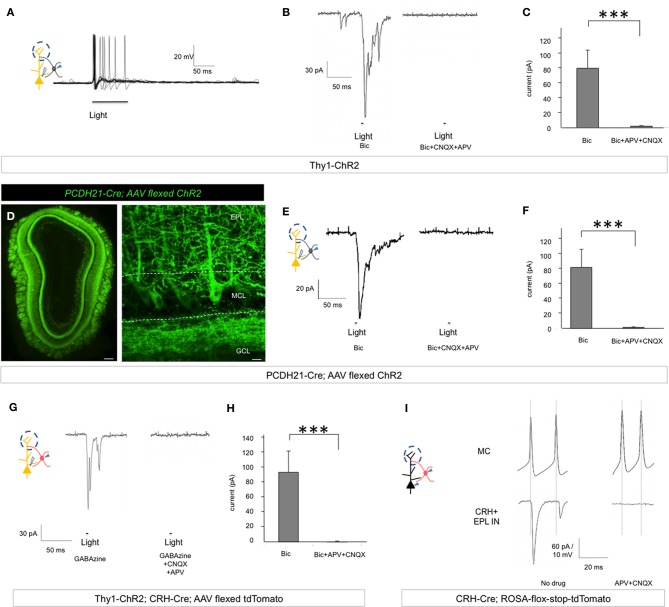
**Mitral cells make excitatory connections onto EPL interneurons. (A)** Postsynaptic firing response and action potential generation in EPL interneurons following light-induced activation of mitral cells. Trace represents an overlay of 10 sweeps from a single recorded EPL from a *Thy1-ChR2* mouse. **(B)** Left, EPL interneuron excitatory postsynaptic current in response to light-activation of mitral cells in the presence of Bicuculline in *Thy1-ChR2* mice. Right, photo-responses can be blocked by APV + CNQX. **(C)** Peak amplitudes of light-evoked postsynaptic currents in EPL interneurons in the presence or absence of glutamatergic blockers in *Thy1-ChR2* mice. Bic, *n* = 9; Bic + APV + CNQX, *n* = 8. Student's *t*-test, ^***^p < 0.01. Bic, Bicuculline. Error bars represent SEM. **(D)** Conditional ChR2 expression 12 d post-injection of an AAV-encoding flexed ChR2 into *PCDH21-Cre* mice. ChR2 is expressed in mitral cells of the olfactory bulb. Left, epifluorescence microscope image, scale bar, 200 μm. Right, confocal microscope image showing a high magnification view of conditional AAV-ChR2 expression selectively in mitral cell membranes. The images are from coronal sections. Scale bar, 20 μm. EPL, external plexiform layer; MCL, mitral cell layer; GCL, granule cell layer. **(E)** Left, EPL interneuron excitatory postsynaptic current in response to light-activation of mitral cells in the presence of Bicuculline in *PCDH21-Cre; AAV flexed ChR2* mice. Right, photo-responses can be blocked by APV + CNQX. **(F)** Peak amplitude of light-evoked postsynaptic currents in EPL interneurons in the presence or absence of glutamatergic blockers in *PCDH21-Cre; AAV flexed ChR2* mice. Bic, *n* = 9; Bic + APV + CNQX, *n* = 7. Student's *t*-test, ^***^p < 0.01. Bic, Bicuculline. Error bars represent SEM. **(G)** Left, CRH+ EPL interneuron excitatory postsynaptic current in response to light-activation of mitral cells in the presence of GABAzine in *Thy1-ChR2; CRH-Cre; AAV flexed tdTomato* mice. Right, photo-responses were blocked by APV + CNQX. **(H)** Peak amplitude of light-evoked postsynaptic currents in CRH+ EPL interneurons in the presence or absence of glutamatergic blockers in *Thy1-ChR2; CRH-Cre; AAV flexed tdTomato* mice. GABAzine, *n* = 9; GABAzine + APV + CNQX, *n* = 7. Student's *t*-test, ^***^p < 0.01. Error bars represent SEM. **(I)** Unitary connection from a mitral cell to a CRH+ EPL interneuron. A short pulse of current was injected to generate action potentials in mitral cells, and strong postsynaptic currents were observed in the CRH+ EPL interneurons (left). The currents were completely blocked by APV + CNQX (right).

Throughout the nervous system, interneurons that act to inhibit principal neurons often receive excitatory input from the same principal neurons to form a negative feedback circuitry (McBain and Fisahn, 2001; Markram et al., 2004). Having shown that EPL interneurons inhibit mitral cells, we next asked whether EPL interneurons and mitral cells exhibit reciprocal connectivity. To investigate this question, we set out to specifically manipulate mitral cell activity while recording EPL interneuron responses. For this, we used two independent approaches. First, we took advantage of *Thy1-ChR2* line 18 mice that express ChR2 in mitral cells of the MOB (Arenkiel et al., 2007; Wang et al., 2007). We made acute olfactory bulb slices and performed whole cell electrophysiology recordings of EPL interneurons to measure postsynaptic responses to mitral cell photostimulation. We observed strong excitation and increased EPL interneuron firing rates in response to mitral cell stimulation (Figure 8A). We then held EPL interneuron potentials at −80 mV and measured their light-evoked postsynaptic currents. With selective mitral cell activation, we observed strong postsynaptic currents in EPL interneurons (94.68 ± 28.19 pA, *n* = 9) in the presence of 50 μM Bicuculline, and these currents were blocked in the presence of 20 μM APV and 20 μM CNQX (2.23 ± 0.86 pA, *n* = 8), suggesting glutamatergic input (Figures 8B,C). Time lantencies from light activation to the onset of the postsynaptic current were 12.50 ± 0.36 ms, which implies direct connectivity from mitral cells to EPL interneurons, given the fact that mitral cell initial spike latency was ~9.9 ms (Arenkiel et al., 2007). Interestingly, we also observed multiple components of EPL interneuron postsynaptic currents arriving at different time latencies in response to photostimulation; even though we cannot fully rule out the possibility that mitral cells also activated EPL interneurons in a polysynaptic manner, it is likely that these postsynaptic current components originated from multiple mitral cells that fired and released neurotransmitter with various time delays.

To corroborate these data, we also utilized a *Protocadherin21-Cre (PCDH21-Cre)* mouse line in conjunction with “flexed” AAV for conditional expression of ChR2 in cells that express Cre (Atasoy et al., 2008). *PCDH21-Cre* mice show restricted Cre expression in mitral cells of the MOB (Nagai et al., 2005). Injecting AAV-encoding flexed ChR2 into olfactory bulbs of *PCDH21-Cre* mice allowed strong and selective expression of ChR2 in mitral cells (Figure 8D). Two weeks post-injection, we made acute brain slices from these mice and performed whole cell voltage clamp recordings from EPL interneurons while photostimulating mitral cells. Similar to the *Thy1-ChR2* transgenic model, we observed strong postsynaptic currents in EPL interneurons in the presence of 50 μM Bicuculline (85.66 ± 22.36 pA, *n* = 9), and these currents were blocked in the presence of 20 μM APV and 20 μM CNQX (2.37 ± 0.79 pA, *n* = 7), suggesting glutamatergic input (Figures 8E,F). Time latencies from light activation to the onset and peak of the postsynaptic current were 11.88 ± 0.81 ms. Together, these data support a model of strong excitatory circuitry from mitral cells to EPL interneurons in the MOB.

Next, to selectively study the connectivity between mitral cells and CRH+ EPL interneurons, we took advantage of *Thy1-ChR2; CRH-Cre* mice and injected a flexed AAV red fluorescent protein reporter for conditional expression of tdTomato in the olfactory bulb. Two weeks post-injection, we prepared acute brain slices from these mice, and performed whole cell patch clamp recordings from tdTomato+ EPL interneurons. Similarly to what we observed in previous experiments, CRH+ EPL interneurons received strong and fast postsynaptic currents in response to mitral cells photostimulation in the presence of 12.5 μM GABAzine (amplitude, 94.68 ± 28.19 pA, *n* = 9; time latency from the light initiation to the onset of postsynaptic current, 12.37 ± 0.50 ms, *n* = 9), which were blocked by 20 μM APV and 20 μM CNQX (2.23 ± 0.86 pA, *n* = 7) (Figures 8G,H).

Having shown that CRH+ EPL interneurons receive strong and fast excitatory input from mitral cells, we next attempted to record paired connections from mitral cells to CRH+ EPL interneurons. In *CRH-Cre; lox-stop-lox-tdTomato* mice, we performed paired whole cell recordings from mitral cells and labeled CRH+ EPL interneurons within distances of 80 μm from each other. Out of 10 recorded pairs, 5 pairs showed strong unitary connectivity (Figure 8I). The average postsynaptic current amplitude was 61.74 ± 21.76 pA, with an average latency of 1.02 ± 0.19 ms from the peak of mitral cell action potentials (*n* = 5, mean ± SEM). The currents were completely blocked by 20 μM APV and 20 μM CNQX. Together, our data support a novel model of CRH+ EPL interneuron—mitral cell circuitry within the MOB: CRH+ EPL interneurons make inhibitory connections onto mitral cells, and receive reciprocal, excitatory input from mitral cells.

## Discussion

**Figure 9:**
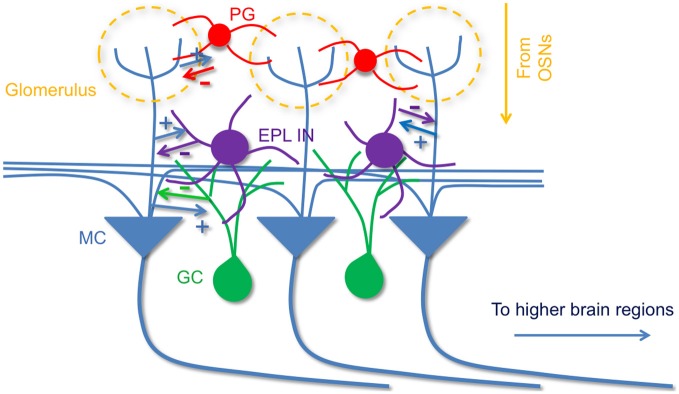
**A model of olfactory bulb circuitry that includes EPL interneurons.** Mitral cells receive odor input from sensory neurons at glomeruli and send output to higher brain areas. Periglomerular cells inhibit mitral cells at their apical dendrites within the GL layer, and local granule cells provide inhibition onto mitral cells via dendrodendritic synapses in EPL layer. In addition to granule cells, we propose that intrinsic EPL interneurons also make reciprocal connections with mitral cells; they receive excitatory input from mitral cells, and send inhibitory feedback to them. The reciprocal connection between EPL interneurons and mitral cells may facilitate critical aspects of olfactory processing. + indicates excitatory connections and, − indicates inhibitory connections.

In prominent models of the MOB circuitry mitral cells are described to receive olfactory information from olfactory sensory neurons, then process and relay this information to higher brain areas, while interneurons in the MOB local circuits sculpt mitral cell activity and output. These interneurons include periglomerular cells (McGann et al., 2005; Shao et al., 2012), superficial short-axon cells (Aungst et al., 2003), and granule cells, which form dendrodendritic synapses with mitral cells to mediate lateral inhibition (Schoppa and Urban, 2003). Here, using cell type-specific optogenetic targeting approaches, we have found that in addition to the mitral cell—granule cell circuitry, EPL interneurons also provide strong inhibition onto mitral cells. Moreover, we have found that EPL interneuron activity is controlled by fast glutamatergic input from mitral cells. Together, our data support the presence of strong and reciprocal connectivity between EPL interneurons and mitral cells in the MOB, which may act in conjunction with other known interneuron types in the bulb to mediate lateral interactions (Figure 9).

### CRH-expressing EPL interneurons

EPL interneurons were originally identified and have been primarily investigated through anatomical and imaging methods (Toida et al., 1994; Kosaka and Kosaka, 2008; Lepousez et al., 2010b; Arenkiel et al., 2011). In search of molecular markers to classify differential subsets of EPL interneurons, previous studies have described EPL interneurons with somatostatin or parvalbumin immunoreactivity (Kosaka and Kosaka, 2008; Lepousez et al., 2010b), but these markers do not show subpopulation-specific restriction in the EPL, and have been shown to localize within other olfactory bulb cell layers. Here we describe for the first time a restricted pattern of CRH expression in a subset of EPL interneurons, and further show that CRH-Cre driver mice allow for selective activity manipulations in this EPL-restricted neuronal population. Using *CRH-Cre* animals, we performed Cre-dependent genetic reporter analysis to identify EPL interneuron subtypes that are marked by CRH, and by functional optogenetic analysis to reveal a reciprocal circuitry between mitral cells and these interneurons on a functional level. Interestingly, only about 30% of EPL interneurons express CRH, which suggests that EPL interneurons may be a group of highly heterogeneous neurons, and/or that CRH-expressing cells may perform a specialized function. Thus, it deserves further investigation to determine if different EPL interneurons are functionally organized within the olfactory bulb circuitry in different ways. For example, tyrosine hydroxylase immunoreactive EPL interneurons have been shown on an anatomical level to lack dendrodentritic synapses with mitral and tufted cells (Liberia et al., 2012). Through combined optogenetic reporter targeting and electrophysiological experimentation we have identified reciprocal connectivity between mitral cells and CRH-expressing EPL interneurons.

In the present study, all experiments were designed to test the synaptic communication between CRH-expressing EPL interneurons and mitral cells. However, we have yet to determine whether CRH secreted by this subpopulation of EPL interneurons actually plays a role in the MOB circuitry. CRH is a small, secreted neuropeptide involved in diverse physiological processes including food intake, stress, reproduction, and fear responses (Schulkin et al., 2005; Gao and Horvath, 2007), all of which are directly influenced by olfactory processing. Furthermore, researchers have shown that in extra-hypothalamic systems, CRH can modulate circuit activity and animal behaviors: in the central nucleus of the amygdala (CeA)—bed nucleus of stria terminalis (BNST) circuitry, CRH can regulate BNST activity and mediate animal fear responses (Schulkin et al., 2005). Thus, it deserves further investigation to determine whether CRH modulates neural activity, mediates neural plasticity, or facilitates olfactory processing together with other known neuromodulators such as dopamine, somatostatin, and/or acetylcholine in the MOB (Ennis et al., 2001; Mechawar et al., 2004; Lepousez et al., 2010a; Ma and Luo, 2012).

### The role of EPL interneurons in olfactory processing

Numerous types of GABAergic interneurons have been identified in the MOB (Batista-Brito et al., 2008; Eyre et al., 2008, 2009; Kosaka and Kosaka, 2011), including granule cells, periglomerular cells, deep short-axon cells, and EPL interneurons. GCs and periglomerular cells have been proposed to mediate diverse functions in olfactory processing, including gain control of mitral cell activity, molecular receptive range (MRR) narrowing and contrast enhancement, synchronizing mitral cell firing timing, or decorrelating mitral cell firing to enhance odor encoding (Yokoi et al., 1995; Schoppa et al., 1998; Laurent, 1999; Luo and Katz, 2001; Aungst et al., 2003; Schoppa and Urban, 2003; Wilson and Mainen, 2006; Arevian et al., 2008; Fantana et al., 2008; Tan et al., 2010). Another interesting feature of these neuronal lineages is that they exhibit adult neurogenesis. In the adult brain, periglomerular cells and granule cells are continuously generated from the stem cells in the subventricular zone; they migrate via the rostral migratory stream, and ultimately integrate into the MOB circuitry (Temple and Alvarez-Buylla, 1999; Lledo and Saghatelyan, 2005; Lledo et al., 2006; Arenkiel et al., 2011), suggesting a highly plastic role in olfaction. Other populations of local interneurons, including Blanes cells and deep short-axon cells (Pressler and Strowbridge, 2006; Eyre et al., 2008, 2009; Arenkiel et al., 2011), provide intrabulbar and extrabulbar GABAergic connections onto local interneurons as well as neurons in higher olfactory areas, but their functional role in olfactory processing remains unknown. Here, we functionally describe for the first time a reciprocal circuitry between EPL interneurons and mitral cells, consistent with previous electron microcopy evidence suggesting the presence of dendrodendritic synapses between mitral cells and a subset of EPL interneurons (Toida et al., 1994; Lepousez et al., 2010b). Based on previous anatomical descriptions, and the functional analysis described here, it is compelling to assume that EPL interneurons are likely to mediate lateral interactions between different glomerular units through lateral inhibition. However, unlike granule cells or periglomerular cells that undergo adult neurogenesis, EPL interneurons may be involved in more stereotyped neural circuit and olfactory functions.

In the future, it will be particularly interesting to elucidate how this synaptic connectivity is organized in three-dimensional space, as well as determine the functional consequences of EPL interneuron-mediated inhibition upon olfactory processing. Further investigations using scanning microscopy to optogenetically stimulate presynaptic neurons (Petreanu et al., 2007; Katzel et al., 2011) while recording from postsynaptic targets will provide a more detailed connectivity map between mitral cells and EPL interneurons, which can in turn advance our knowledge of the connectivity and function of the MOB circuitry. Ultimately, higher resolution mapping experiments will be required to reveal the detailed spatial organization of the input and output of these neurons. By investigating the topographic organization of bulbar connectivity, we will gain further insight into the different roles for interneuron subtypes in olfaction, as well as elucidate basic mechanisms that underlie microcircuit processing.

## Conflict of interest statement

The authors declare that the research was conducted in the absence of any commercial or financial relationships that could be construed as a potential conflict of interest.
